# Influences of the COVID-19 Pandemic on Obesity and Weight-Related Behaviors among Chinese Children: A Multi-Center Longitudinal Study

**DOI:** 10.3390/nu14183744

**Published:** 2022-09-10

**Authors:** Yirong He, Biru Luo, Li Zhao, Shujuan Liao

**Affiliations:** 1 Department of Nursing, West China Second University Hospital, Sichuan University, Chengdu 610041, China; 2 Key Laboratory of Birth Defects and Related Diseases of Women and Children, Sichuan University, Ministry of Education, Chengdu 610041, China; 3 Department of Health Policy and Management, West China School of Public Health/West China Fourth Hospital, Sichuan University, Chengdu 610041, China

**Keywords:** COVID-19, pediatric obesity, life style, longitudinal studies

## Abstract

COVID-19-related lockdown measures have been affecting children’s weight status and weight-related behaviors, which are often associated with an increase in childhood obesity. However, large-scale longitudinal studies are lacking. Our study aimed to analyze changes in obesity and weight-related behaviors in Chinese children before and during the COVID-19 pandemic and provide references for addressing the high prevalence of childhood obesity. A prospective multi-center longitudinal survey was conducted among Chinese children (*n* = 5963), collecting data on weight status, COVID-19-related measures, and lifestyle behaviors. Changes were assessed using t-tests and χ^2^ tests for paired samples, or the Wilcoxon signed-rank test, according to the type and distribution of data. The Generalized Estimating Equations model was used to explore influential factors of obesity. The prevalence of overweight and obesity increased from 9.2% and 8.6% before the pandemic to 10.5% and 10.6% during the pandemic (*p* < 0.001), respectively. Daily physical activity, sleep duration, and sugar-sweetened beverage consumption decreased while screen time increased. The results of the generalized estimating equations showed that ethnic minority, older age, less daily physical activity, reduced sleep duration, and longer screen time were positively associated with obesity. There is an intensifying trend of obesity in children in the context of the COVID-19 pandemic, to which altered weight-related behaviors might have contributed largely. Maintaining a healthy lifestyle, especially in social crises, should be highlighted to ease the burden of childhood obesity.

## 1. Introduction

Obesity is particularly prominent among the various health problems faced during children’s growth and development [1,2]. The high prevalence of childhood obesity has become a global health challenge [3], and it is increasing worldwide [4]. The prevalence of overweight and obesity is reported to be 11.1% and 7.9%, respectively, in Chinese children aged 6 to 17 years [5]. Overweight and obesity in childhood are associated with adverse health outcomes [6], including psychological problems, asthma, obstructive sleep apnea, orthopedic problems, and adverse cardiovascular disease [7]. Moreover, obesity in childhood may persist into adulthood and lead to adverse cardiovascular outcomes or other obesity-related diseases [8]. Previous studies have disclosed a variety of factors that are associated with childhood obesity, such as unhealthy lifestyle, genetics, environment, and metabolism [9]. Among these, lifestyle is a reversible factor that contributes largely to overweight and obesity; thus, a variety of measures have been implemented to improve healthy lifestyles to alleviate the impact of childhood obesity [10]. 

The fast spread of COVID-19, which first broke out at the end of 2019, has become a global pandemic, posing a serious threat to people’s physical and mental health and socioeconomic development [11]. To curb the spread of COVID-19, strict social isolation measures have been implemented by many countries, including lockdown, quarantines, and school and business closures [12]. In Sichuan, China, as of July 2020, 604 COVID-19 cases had been confirmed. Schools closed from March 2020, with more than one million students studying online [13]. Social restrictions and online learning might have interrupted children’s regular lifestyle and weight-related behaviors, such as physical activity, screen time, sleep duration, and eating behavior [14], and exacerbated the problem of childhood obesity by undermining well-functioning weight control measures.

Researchers have noticed this inevitable problem. Evidence from a cross-sectional study shows increased body mass index and obesity prevalence among adolescents due to lockdown policies under the COVID-19 epidemic [15]. Studies focusing on lifestyles found that children’s sleep duration and screen time increased, while their physical activity decreased [16,17,18]. However, most of the published studies assessing the impact of the COVID-19 pandemic on obesity and weight-related behaviors were cross-sectional and were conducted during the initial months of the pandemic [19,20], leaving a knowledge gap regarding the exact influence of the pandemic, as well as hindering us from better understanding the whole picture of variation trends of childhood obesity amid this specific social crisis.

Therefore, this study adopted a two-wave longitudinal design, aiming to analyze the changes in obesity and weight-related behaviors of Chinese children before and during the COVID-19 pandemic and explore their longitudinal relationships. Our findings provide reference information for global strategies and policies to address the impact of social crises such as the COVID-19 pandemic on childhood obesity.

## 2. Materials and Methods

### 2.1. Study Design and Participants

This study was a prospective multi-center longitudinal study that explored the changes in obesity and weight-related behaviors among children before and during the COVID-19 pandemic. The data were collected from the Chengdu Positive Child Development (CPCD) survey [21]. A total of 6900 students were recruited from 5 primary and secondary schools in Chengdu by cluster sampling and took part in the first wave assessment. In total, 5963 participants completed the entire study, with an overall response rate of 86.4%. There were no significant differences in sociodemographic characteristics between participants who completed the final survey and those who were lost to follow-up. The two waves of data were collected in the classroom setting in an onsite manner. The first wave of data was collected before the pandemic (from 23 December 2019 to 13 January 2020). The second wave of data was collected during the pandemic (from 16 June 2020 to 8 July 2020), when the epidemic subsided and schools reopened in China. Data were collected by trained researchers using the same instructions. All participants completed questionnaires independently, and the completed questionnaires were returned immediately to the researchers.

The study complied with the Declaration of Helsinki and was approved by the Ethics Committee of Sichuan University with registration number K2020025. All individuals volunteered to participate in the study with written informed consent, and strict confidentiality was guaranteed. Participants had the right to withdraw at any stage of the study.

### 2.2. Measures and Procedure

#### 2.2.1. Weight Status Variables

Weight status variables included height and weight, which were collected at both waves. In wave 1, children’s height and weight were measured by professional technicians from community hospitals. All investigators and medical examiners involved in the study entered the schools after a unified training program. All height and weight measurement instruments adopted a unified brand, and participants were selected for quality control in a randomized manner at a rate of 5% during the daily testing process. An error of 0.5 cm is allowed for height and 0.1 kg for weight. If the incidence of error is >5% per day, the reasons and methods of improvement should be investigated; if the incidence is >10%, the data for that day are invalid, and re-testing is carried out. Boys and girls were measured separately and were asked to stand upright during the measurement with their shoes and hats off, heels together, and heels, sacrum, and shoulder blades in contact with the column. To measure height, we used a rangefinder to measure standing height to the nearest 0.1 cm. To measure weight, we used a precise digital scale to the nearest 0.1 kg. Each participant’s physical examination form has its own code. Due to the crowd gathering limitation implemented to constrain the COVID-19 epidemic in wave 2, children’s height and weight were reported by their parents.

Overweight and obesity are defined based on body mass index (BMI), which is calculated by dividing weight in kilograms by the square of height in meters. Participants were assessed for weight status based on age- and sex-specific BMI thresholds in the World Health Organization (WHO) growth curve for children [22]. 

#### 2.2.2. Weight-Related Behaviors

Participants’ weight-related behaviors were measured onsite in both waves using a self-developed form. The form was completed by the participants independently after explanation by the researchers. Investigators collected filled forms and checked their accuracy and completeness. The form assessed participants’ physical activity, sleep duration, screen time, and eating behaviors (food purchase, dining habits, staple food intake, fruit and vegetable intake, and sugar-sweetened beverage consumption). Participants were asked to fill out the form based on their experiences of the past week. 

#### 2.2.3. Demographics

Participants’ demographic information was measured in wave 1 using a self-developed form. Demographic information included seven items: gender (boy and girl), age (years), ethnicity (Han and minorities), district (urban and rural), education (primary school and junior high school), pocket money (0–10 yuan, 11–30 yuan, and >30 yuan), and household income (<1000 yuan, ≥1000–5000 yuan, ≥5000–10,000 yuan, ≥10,000–20,000 yuan, ≥20,000 yuan). COVID-19 infection history (“Did you or your family members get infected by COVID-19?”) was collected in wave 2. The procedure of data collection was in line with that mentioned above. 

### 2.3. Data Analysis

Statistical analysis was conducted by SPSS 26.0(IBM Corp., Armonk, NY, USA). Normal numerical variables were expressed as mean ± SD and non-normal numerical variables were expressed as median (P_25_–P_75_). The categorical variables were expressed as *n* (%). Changes in BMI, prevalence of overweight and obesity, physical activity, sleep duration, screen time, and eating behavior at the two waves were assessed using *t*-tests and *χ*^2^ tests for paired samples, or Wilcoxon signed-rank tests, according to the type and distribution of data [23]. The Generalized Estimating Equations model (GEE, using SPSS software, version 26.0) was used to identify variables that contributed to the alteration in obesity in the two waves. All statistical tests were two-sided, and a *p* value of less than 0.05 was considered statistically significant.

## 3. Results

### 3.1. Demographics

Table 1 presents the baseline characteristics of our sample. A total of 5963 participants were included in the final analysis. Of the total, 2973 (49.9%) of our participants were boys and 2990 (50.1%) were girls. The mean age of the participants was 10.7 ± 2.2 years. The majority of our participants were Han (99.2%), in primary school (80.6%), and from urban areas (65.8%). More than half of the participants (67.1%) had pocket money of 0–10 yuan per month, and nearly two-fifths of them (42.1%) had a household income of 5000–10,000 yuan per month. The average BMI of children was 18.4 ± 3.2 kg/m^2^, 9.2% of them were overweight, and 8.6% were obese.

### 3.2. Changes in Weight Status

As summarized in Table 2, the average BMI increased from 18.4 ± 3.2 kg/m^2^ to 18.5 ± 3.2 kg/m^2^ (*p* < 0.05), and the increase in BMI was greater for boys than girls. Participants who were Han and lived in urban areas had a significant increase in BMI (*p* < 0.001). The BMI of students of all age groups increased significantly (*p* < 0.001). Participants’ weekly pocket money was divided into three classes (0–10 yuan, 11–30 yuan, and greater than 30 yuan), and an increasing trend in BMI was found in each subgroup with a *p* value of less than 0.05. Participants whose monthly household income was between 1000 and 5000 yuan showed a significant increase in BMI compared to others (18.3 to 18.5 kg/m^2^, *p* < 0.001).

The prevalence of overweight in children increased from 9.2% to 13.0% (*p* < 0.001), and the obesity prevalence increased from 8.6% to 10.6% (*p* < 0.001) (Table 2). The prevalence of overweight and obesity increased among boys but not girls. There was a significant increase in the prevalence of overweight and obesity among children in rural areas and junior high schools. Similarly, increases in the prevalence of obesity were also observed in children with pocket money of less than 30 yuan per month. Compared with the pre-pandemic session, an increase in overweight was found in families with a monthly income of 1000–5000 yuan and more than 20,000 yuan, while the prevalence of obesity increased in children with a monthly income of 5000–20,000 yuan.

### 3.3. Changes in Weight-Related Behaviors 

Changes in weight-related behaviors are reported in Table 3. Participants’ time spent on daily physical activity decreased from 90.4 ± 50.9 min before to 83.1 ± 50.6 min during the pandemic (*p* < 0.001). Similarly, participants’ sleep duration decreased from 549.7 ± 65.0 min to 534.6 ± 71.8 min (*p* < 0.001). The screen time (time spent on electronic devices such as TV, mobile phones, pads, and computers for entertainment per day) of our participants increased from 85.3 ± 74.5 min to 100.1 ± 73.9 min (*p* < 0.001). The frequency of weekly sugar-sweetened beverage consumption decreased from 1.5 ± 1.9 to 1.1 ± 1.7 times (*p* < 0.001). 

The results of subgroup analysis demonstrated that the physical activity and sleep duration of participants decreased significantly from wave 1 to wave 2 in each subgroup (*p* < 0.05), except for ethnic minorities. Similarly, the screen time increased significantly (*p* < 0.05) from before to during the pandemic in each subgroup, except for participants with a household income of less than 1000 yuan/month and ethnic minority groups. In all subgroups, the frequency of weekly sugar-sweetened beverage consumption was significantly reduced (*p* < 0.05) at wave 2 compared to wave 1.

### 3.4. Influencing Factors of Childhood Obesity

We used the GEE model with exchangeable working correlation matrix, linear regression, and main effects models to explore the variables that may contribute to changes in BMI. Participants’ demographic data, history of COVID-19 infection, physical activity, sleep duration, screen time, and sugary beverage consumption were input into the GEE model as factors (categorical data) and covariables (numerical data). The results of the generalized estimating equations are shown in Table 4. Han ethnicity (*β* = −0.882, *p* = 0.002), primary school (*β* = −1.684, *p* < 0.001), COVID-19 infection history (*β* = −4.060, *p* < 0.001), time point wave 1 (*β* = −0.064, *p* = 0.043), longer physical activity duration (*β* = −0.001, *p* = 0.018), and longer sleep duration (*β* = −0.002, *p* < 0.001) were the protective factors to reduce childhood obesity. Older age (*β* = 1.060, *p* < 0.001) and longer screen time (*β* = 0.001, *p* < 0.001) were the risk factors identified.

## 4. Discussion

This prospective multi-center longitudinal study investigated changes in the weight status and weight-related behavior of children in China before and during the COVID-19 pandemic. The results of the study showed an increase in BMI and prevalence of overweight and obesity in children during the COVID-19 pandemic compared with before, particularly among boys. This finding is in line with previous reports. A longitudinal study in Israel found that the overall prevalence of obesity increased by 1.8% after the pandemic [24]. A U.S. survey of 191,509 school-age youth regarding associations between weight and the pandemic showed that youths gained more weight during the COVID-19 pandemic than before, and overweight or obesity increased among 5–11-year-olds from 36.2% to 45.7% during the pandemic [25]. The results of this study also suggested that the prevalence of overweight and obesity among children in rural areas increased significantly during the pandemic. The intact self-sufficient food supply and inactive lifestyle might be the reasons [26]. The prevalence of overweight and obesity among children in junior high school increased significantly compared with those in primary school. This may be because junior high school students had greater stress concerning their learning, and their exercise was relatively at a lower level [27]. Family economic factors also have a noticeable relationship with childhood obesity [28]. The higher the monthly family income, the more likely children are to suffer from obesity [29]. These additional and interesting findings of the current study enriched our understanding of childhood obesity during the COVID-19 pandemic, and shed light on further research directions to explore the influences of sociodemographic background on weight status. 

Since the outbreak of COVID-19, many countries have implemented strict measures to limit avoidable morbidity and mortality. These include complete or partial lockdown, closing schools and public places, etc. [30]. During the lockdown period, children studied online at home. All these social isolation measures may have negative impacts on weight status and lifestyles [14]. In line with previous studies, our findings stress the necessity and importance of weight management for children in the context of the COVID-19 pandemic. This study found that the COVID-19 pandemic had changed children’s weight-related behaviors, which may contribute to obesity. Daily physical activity and sleep duration decreased in our sample, while screen time increased. An online survey of high school, university, and graduate students in China found changes in activity patterns among adolescents under isolation measures, with a significant decrease in the frequency of leisure-time physical activity and increased screen time [31]. In the MUGI project conducted with a cohort of Spanish children, the results shed light on future consequences for children’s health due to strict confinement, showing a significant change in physical activity levels (−91 min/d) and screen time (+1.8 h/d) during the COVID-19 confinement [32]. Similar to our findings, another study conducted in the Spanish population reported that 79.2% of participants referred to delayed bedtime and 16.3% of participants were suspected of having sleeping disorders after the implementation of lockdown [33]. Taken together, these findings emphasize the negative influences of the COVID-19 pandemic on health-related behaviors among children, and appeal to efficient interventions. 

In addition, our results of generalized estimating equation analysis show that children with lower levels of physical activity, shorter sleep duration, and longer screen time are more likely to be obese. During the COVID-19 pandemic, children had reduced access to regular, mandatory physical activity at school and lacked public places to exercise, which may have led to a reduction in physical activity [34,35]. During home isolation, children lost their unified school schedule, which may have led to irregular sleep patterns [36]. The reason underlying the decrease in sleep duration might be the increased screen time, as nighttime exposure to bright light suppresses melatonin production [37]. Children are more likely to be obese if they spend more time at home watching TV or using mobile phones, tablets, computers, and other electronic products for entertainment. Many studies have reported the relationship between screen usage and obesity. The obesity caused by using screens for a long time may be due to the calorie intake [38]. For example, watching TV while eating may increase the calorie intake by delaying the satiety during eating or reducing the satiety signal of previously ingested food [39]. Additional longer screen time associated with increased sedentary behavior and decreased physical activity may also contribute to obesity [40,41]. Therefore, enough physical activity, sleep duration, and controlled screen time are important factors in reducing the prevalence of obesity in children during the COVID-19 pandemic.

### Strengths and Limitations

This study presents exclusive advantages in accumulating a large and timely sample size in the pediatric population, which is particularly important in the context of public health emergencies. This longitudinal study also extends previously published cross-sectional studies that provided insight into the long-term impact of the pandemic on childhood obesity. The information provided by this study should be useful to policy makers, school administrators, and parents to understand the current status of childhood obesity and changes in weight-related behaviors among Chinese children to take measures to minimize adverse changes.

However, several limitations should be acknowledged. First, the height and weight of the participants in wave 2 were reported by their parents rather than measured by professionals due to the COVID-19 pandemic. It was not possible to determine whether and how participants measured their height and weight, which may result in underestimated or exaggerated results. Second, the content of the survey on lifestyle is relatively simple, with only one item for each activity. More comprehensive measurement tools need to be considered in future studies. Third, although the selection of research objects adopts the principle of cluster sampling, the research samples are limited to Sichuan Province, which may weaken the representativeness of our findings. Participants from other parts of China should also be considered in further research. Fourth, although the longitudinal design of the current study, it may be difficult to establish a clear causal relationship from this observational study. Fifth, there may be other confounding factors that alter the associations we find or produce “false” associations. Additional confounding variables such as genetics, season, weather, physical condition, parental education, living arrangements, and social environment may need to be considered to enhance the robustness of the longitudinal association found in this study.

## 5. Conclusions

In summary, our study findings suggest that there is an intensifying trend of obesity in children in the context of the COVID-19 pandemic, to which altered weight-related behaviors might have contributed largely. Although measures taken to mitigate the pandemic are essential to protect public health, they have a negative impact on the weight status and lifestyle of children. Therefore, maintaining a healthy lifestyle, especially during any social crisis such as the COVID-19 pandemic, should be emphasized to ease the burden of childhood obesity.

## Figures and Tables

**Table 1:** Participants’ baseline characteristics.

	Boys	Girls	Included Sample	Excluded Sample
Variable	(*n* = 2973)	(*n* = 2990)	(*n* = 5963)	(*n* = 937)
Age (years)	10.6 ± 2.2	10.6 ± 2.2	10.7 ± 2.2	10.8 ± 1.7
Ethnicity				
Han	2946(99.1)	2969(99.3)	5915(99.2)	928(99.1)
Minority	27(0.9)	21(0.7)	48(0.8)	9(0.9)
District				
Urban	1975(66.4)	1948(65.2)	3923(65.8)	618(66.0)
Rural	998(33.6)	1042(34.8)	2040(34.2)	319(34.0)
Education				
Primary school	2042(68.7)	1960(65.6)	4002(67.1)	642(68.5)
Junior high school	931(31.3)	1030(34.4)	1961(32.9)	295(31.5)
Pocket money (yuan/mouth)				
0–10	1445(48.6)	1501(50.2)	2946(49.4)	469(50.1)
11–30	1025(34.5)	1007(33.7)	2032(34.1)	317(33.8)
>30	503(16.9)	482(16.1)	985(16.5)	151(16.1)
Household income(yuan/mouth)				
<1000	50(1.7)	45(1.5)	95(1.6)	19(2.0)
≥1000–5000	552(18.5)	568(19.0)	1120(18.9)	174(18.6)
≥5000–10,000	1242(41.8)	1272(42.5)	2514(42.1)	403(43.0)
≥10,000–20,000	777(26.2)	753(25.2)	1530(25.6)	239(25.5)
≥20,000	352(11.8)	352(11.8)	704(11.8)	102(10.9)
Father’s career				
Farmer	264(8.9)	314(10.5)	578(9.7)	97(10.3)
Worker	401(13.5)	464(15.5)	865(14.5)	141(15.1)
Merchant	247(8.3)	278(9.3)	525(8.8)	80(8.5)
Public servant	668(22.4)	578(19.3)	1246(20.9)	185(19.8)
Office clerk	510(17.2)	468(15.7)	978(16.4)	170(18.1)
Technical personnel	738(24.8)	723(24.2)	1461(24.5)	217(23.2)
Retired	91(3.1)	124(4.1)	215(3.6)	31(3.3)
Unemployed	54(1.8)	41(1.4)	95(1.6)	16(1.7)
Mother’s career				
Farmer	319(10.7)	361(12.1)	680(11.4)	98(10.5)
Worker	333(11.2)	323(10.8)	656(11.0)	110(11.7)
Merchant	356(12.0)	300(10.0)	656(11.0)	93(10.0)
Public servant	599(20.1)	611(20.4)	1210(20.3)	199(21.2)
Office clerk	550(18.5)	547(18.3)	1097(18.4)	167(17.8)
Technical personnel	652(22.0)	642(21.5)	1294(21.7)	211(22.5)
Retired	96(3.2)	107(3.6)	203(3.4)	26(2.8)
Unemployed	68(2.3)	99(3.3)	167(2.8)	33(3.5)
Body composition				
Height (cm)	142.6 ± 13.5	142.4 ± 13.7	142.5 ± 13.6	142.1 ± 14.1
Weight (kg)	39.9 ± 10.6	40.7 ± 11.1	40.3 ± 10.9	41.3 ± 9.4
BMI (kg/m^2^)	18.4 ± 3.2	18.4 ± 3.1	18.4 ± 3.2	18.6 ± 2.9
SBP (mmHg)	103.2 ± 14.7	102.6 ± 14.1	102.9 ± 14.4	103.0 ± 13.9
DBP (mmHg)	71.1 ± 15.8	71.6 ± 15.9	71.4 ± 15.7	71.2 ± 15.3
BMI categories				
Underweight	42(1.4)	15(0.5)	57(0.9)	11(1.2)
Normal weight	2383(80.2)	2465(82.4)	4848(81.3)	756(80.7)
Overweight	302(10.1)	244(8.2)	546(9.2)	90(9.6)
Obesity	246(8.3)	266(8.9)	512(8.6)	80(8.5)

Age and body composition are reported as mean (SD). Ethnicity, district, education, pocket money, household income, parents’ career and BMI categories are reported as *n* (%). BMI: body mass index. SBP: systolic blood pressure. DBP: diastolic blood pressure.

**Table 2:** Changes in weight status of children before and during the pandemic.

		BMI			Prevalence of Overweight			Prevalence of Obesity		
	*N*	2019	2020	*p* ^1^	2019	2020	*p* ^2^	2019	2020	*p* ^3^
Total	5963	18.4 ± 3.2	18.5 ± 3.2	0.013	546(9.2)	627(10.5)	<0.001	512(8.6)	634(10.6)	<0.001
Gender										
Boys	2973	18.4 ± 3.2	18.5 ± 3.3	<0.001	302(10.1)	370(12.4)	0.005	246(8.3)	359(12.1)	<0.001
Girls	2990	18.4 ± 3.2	18.4 ± 3.2	0.730	244(8.2)	257(8.6)	0.544	266(8.9)	275(9.2)	0.685
Ethnicity										
Han	5915	18.4 ± 3.2	18.5 ± 3.2	0.014	539(9.1)	622(10.5)	0.010	502(8.5)	623(10.5)	<0.001
Minority	48	19.3 ± 4.2	19.3 ± 4.3	0.892	7(14.6)	5(10.4)	0.537	10(20.8)	11(22.9)	0.847
District										
Urban	3923	18.4 ± 3.2	18.5 ± 3.2	0.012	377(9.6)	410(10.5)	0.215	203(4.7)	219(5.6)	0.423
Rural	2040	18.5 ± 3.2	18.4 ± 3.2	0.432	169(8.3)	217(10.6)	0.010	309(15.1)	415(24.5)	<0.001
Education										
Primary school	4002	17.2 ± 2.3	17.2 ± 2.4	0.055	240(6.0)	245(6.1)	0.815	273(6.8)	286(7.1)	0.569
Junior high school	1961	20.9 ± 3.5	21.0 ± 3.1	0.116	306(15.6)	382(19.5)	<0.001	239(12.2)	348(23.5)	<0.001
Pocket money (yuan/mouth)										
0–10	2946	18.0 ± 3.2	18.3 ± 3.1	<0.001	236(8.0)	269(9.1)	0.125	225(7.6)	277(9.4)	0.015
11–30	2032	18.7 ± 3.3	19.0 ± 3.2	<0.001	247(12.1)	280(13.8)	0.123	213(10.5)	267(14.6)	0.009
>30	985	18.2 ± 3.1	18.5 ± 3.1	<0.001	63(6.4)	78(8.0)	0.190	74(7.5)	90(9.1)	0.192
Household income (yuan/mouth)										
<1000	95	18.2 ± 3.1	18.3 ± 3.3	0.539	6(6.3)	11(11.6)	0.204	11(11.6)	10(10.5)	0.817
≥1000–5000	1120	18.3 ± 3.2	18.5 ± 3.2	<0.001	84(7.5)	114(10.2)	0.026	86(7.7)	106(9.5)	0.131
≥5000–10,000	2514	18.4 ± 3.3	18.5 ± 3.2	0.142	261(10.4)	257(10.2)	0.853	218(8.7)	270(10.7)	0.013
≥10,000–20,000	1530	18.4 ± 3.2	18.4 ± 3.2	0.418	138(9.0)	161(10.5)	0.161	133(8.7)	164(10.7)	<0.001
≥20,000	704	18.4 ± 3.1	18.5 ± 3.2	0.294	57(8.1)	84(11.9)	0.017	64(9.1)	84(11.9)	0.082

BMI is reported as mean (SD). Prevalence of overweight and prevalence of obesity are reported as *n* (%).^1^ *p* value for paired t-tests of changes in BMI. ^2^ *p* value for paired χ^2^ tests of changes in overweight prevalence. ^3^ *p* value for paired χ^2^ tests of changes in obesity prevalence.

**Table 3:** Changes in weight-related behaviors before and during the pandemic.

		Physical Activity			Sleep Duration			Screen Time			Sugar-Sweetened Beverage Consumption		
	*N*	2019	2020	*p*	2019	2020	*p*	2019	2020	*p*	2019	2020	*p*
Total	5963	90.4 ± 50.9	83.1 ± 50.6	<0.001	549.7 ± 65.0	534.6 ± 71.8	<0.001	85.3 ± 74.5	100.1 ± 73.9	<0.001	1.5 ± 1.9	1.1 ± 1.7	<0.001
Gender													
Boys	2973	90.2 ± 50.8	83.9 ± 51.9	<0.001	548.4 ± 65.7	535.8 ± 72.6	<0.001	87.8 ± 76.0	100.3 ± 73.6	<0.001	1.5 ± 1.9	1.1 ± 1.6	<0.001
Girls	2990	90.6 ± 51.1	82.4 ± 49.4	<0.001	551.1 ± 64.2	533.5 ± 71.0	<0.001	82.9 ± 73.0	99.9 ± 74.1	<0.001	1.5 ± 1.8	1.1 ± 1.7	<0.001
Ethnicity													
Han	5915	90.4 ± 50.8	83.1 ± 50.6	<0.001	549.7 ± 65.0	534.6 ± 71.8	<0.001	85.5 ± 74.5	100.1 ± 73.9	<0.001	1.5 ± 1.9	1.1 ± 1.7	<0.001
Minority	48	93.2 ± 67.3	88.3 ± 54.2	0.605	556.0 ± 60.4	543.6 ± 70.7	0.249	68.3 ± 71.4	92.8 ± 71.7	0.053	1.6 ± 1.8	1.1 ± 1.6	0.005
District													
Urban	3923	90.7 ± 51.2	83.2 ± 51.1	<0.001	549.5 ± 65.4	534.4 ± 71.9	<0.001	80.5 ± 72.9	93.9 ± 71.5	<0.001	1.5 ± 1.8	1.1 ± 1.7	<0.001
Rural	2040	89.7 ± 50.5	83.1 ± 49.7	<0.001	550.2 ± 64.1	535.1 ± 71.6	<0.001	94.6 ± 76.7	111.9 ± 76.8	<0.001	1.7 ± 2.1	1.2 ± 1.7	<0.001
Education													
Primary school	4002	90.8 ± 50.7	83.7 ± 50.8	<0.001	551.6 ± 64.8	537.8 ± 71.4	<0.001	83.4 ± 74.1	97.3 ± 72.9	<0.001	1.5 ± 1.8	1.1 ± 1.6	<0.001
Junior high school	1961	89.6 ± 51.4	82.0 ± 50.3	<0.001	546.0 ± 65.1	528.2 ± 72.2	<0.001	89.2 ± 75.4	105.7 ± 75.5	<0.001	1.6 ± 2.1	1.2 ± 1.8	<0.001
Pocket money (yuan/mouth)													
0–10	2946	92.3 ± 51.7	83.6 ± 51.1	<0.001	556.0 ± 63.5	541.6 ± 70.7	<0.001	85.2 ± 75.0	100.2 ± 73.5	<0.001	1.5 ± 1.8	1.1 ± 1.6	<0.001
11–30	2032	88.2 ± 50.2	82.6 ± 49.3	<0.001	543.3 ± 65.7	526.3 ± 71.4	<0.001	84.7 ± 74.7	100.4 ± 75.5	<0.001	1.5 ± 1.9	1.1 ± 1.6	<0.001
>30	985	89.3 ± 50.0	82.7 ± 51.9	<0.001	544.3 ± 66.1	531.2 ± 73.8	<0.001	86.9 ± 73.1	98.7 ± 71.5	<0.001	1.5 ± 2.0	1.2 ± 1.9	<0.001
Household income (yuan/mouth)													
<1000	95	83.3 ± 51.2	73.3 ± 52.9	0.048	548.1 ± 69.5	530.5 ± 69.0	0.013	88.8 ± 79.2	104.2 ± 82.9	0.066	1.7 ± 2.7	1.1 ± 1.6	0.010
≥1000–5000	1120	90.5 ± 50.9	84.5 ± 49.8	<0.001	548.5 ± 63.7	531.3 ± 72.9	<0.001	85.9 ± 76.1	98.8 ± 74.4	<0.001	1.5 ± 1.8	1.1 ± 1.7	<0.001
≥5000–10,000	2514	90.9 ± 51.5	83.4 ± 51.6	<0.001	547.8 ± 64.8	534.6 ± 70.7	<0.001	83.8 ± 73.3	99.8 ± 73.3	<0.001	1.5 ± 1.8	1.1 ± 1.6	<0.001
≥10,000–20,000	1530	89.5 ± 48.9	82.1 ± 49.7	<0.001	552.6 ± 67.0	536.5 ± 73.2	<0.001	89.1 ± 76.7	101.9 ± 73.8	<0.001	1.5 ± 1.9	1.1 ± 1.6	<0.001
≥20,000	704	91.3 ± 53.2	83.4 ± 50.1	<0.001	552.7 ± 62.0	536.4 ± 71.4	<0.001	81.1 ± 70.7	98.6 ± 74.2	<0.001	1.6 ± 2.1	1.2 ± 1.8	<0.001

All results are reported as mean (SD). *p* value for paired *t*-tests or Wilcoxon signed-rank tests of changes in weight-related behaviors.

**Table 4:** Influencing factors of obesity in children.

Variables	*β*	SE	Wald *χ*^2^	*p*	OR
Gender = boy	−0.071	0.041	3.083	0.079	0.966
Ethnicity = Han	−0.882	0.278	10.072	0.002	0.451
District = urban	−0.030	0.044	0.462	0.497	0.989
Education = primary school	−1.684	0.062	749.309	<0.001	0.186
Infection history = yes	−4.060	0.463	76.768	<0.001	0.017
Time point = wave 1	−0.064	0.031	4.109	0.043	0.938
Age	1.060	0.011	8593.779	<0.001	2.187
Physical activity	−0.001	0.001	5.564	0.018	0.999
Sleep duration	−0.002	0.001	38.032	<0.001	0.998
Screen time	0.001	0.000	30.473	<0.001	1.001
Sugar-sweetened beverage consumption	0.009	0.011	0.593	0.441	1.004

SE: standard error. OR: odds ratio.

## Data Availability

The data presented in this study are available upon request from the corresponding author.
